# Supplementary material for: Dectin-1 as a Potential Inflammatory Biomarker for Metabolic Inflammation in Adipose Tissue of Individuals with Obesity
Source: Cells. 2022 Sep 15;11(18):2879. doi: 10.3390/cells11182879 (PMC9496833; doi:10.3390/cells11182879)
Supplement: Supplementary file 1 [file cells-11-02879-s001.zip › cells-1860922-supplementary.pdf]

cells MDPI

Supplementary Data

Supplementary Figure S1. Dectin-1 antibody specificity validation using spleen tissues. Primary antibody [1:100 dilution of rabbit polyclonal anti-dectin-1 antibody (Abcam, Waltham, MA, USA; #ab140039] and secondary antibody goat anti-rabbit conjugated with horseradish peroxidase polymer chain DAKO EnVision Kit (Dako, Denmark).

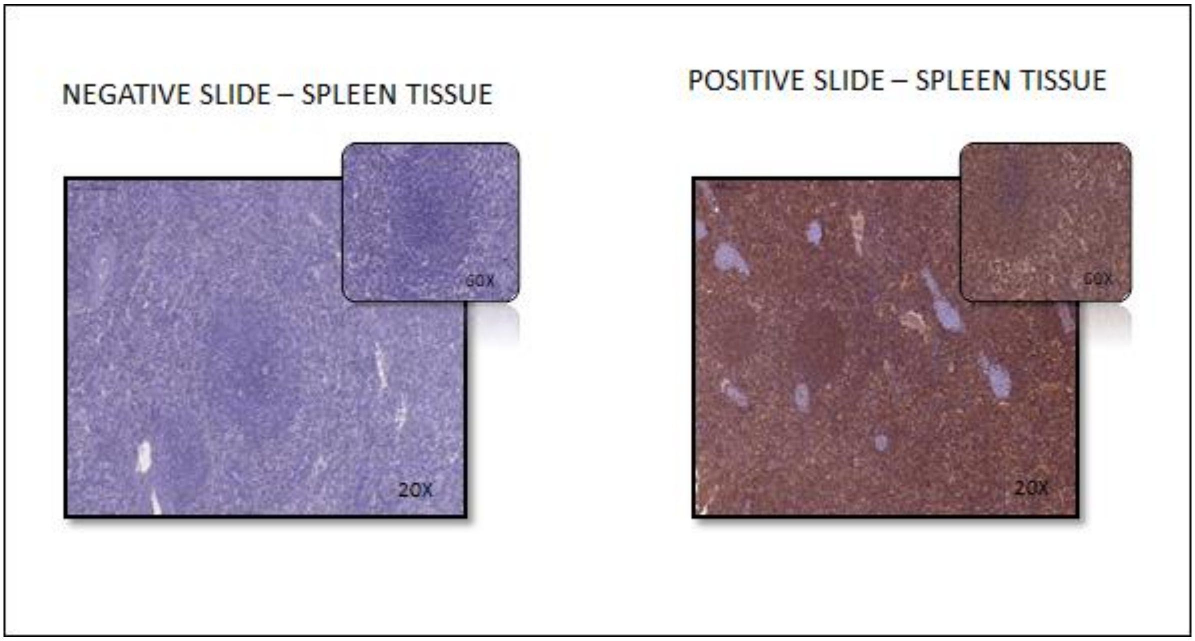

Supplementary Table S1: List of TaqMan Primers used for RT-PCR Assay

Primer List	Assay ID	Primer List	Assay ID
IL-1β	Hs01555410_m1	CCR1	Hs00928897_s1
IL-2	Hs00174114_m1	CCR2	Hs00704702_s1
IL-5	Hs01548712_g1	CCR5	Hs99999149_s1
IL-6	Hs00985639_m1	CXCL9	Hs00171065_m1
IL-8	Hs00174103_m1	CXCL10	Hs01124251_g1
IL-10	Hs00961622_m1	CXCL11	Hs04187682_g1
IL-12A	Hs01073447_m1	CD16	Hs04334165_m1
IL-13	Hs00174379_m1	CD68	Hs02836816_g1
IL-18	Hs01038788_m1	CD86	Hs01567026_m1
IL-23A	Hs00900828_g1	CD163	Hs00174705_m1
IL-33	Hs00369211_m1	TLR2	Hs01872448_s1
TNFα	Hs01113624_g1	TLR3	Hs01551078_m1
CCL2	Hs00234140_m1	TLR4	Hs00152939_m1
CCL3	Hs04194942_s1	TLR7	Hs01933259_s1
CCL5	Hs00982282_m1	TLR8	Hs00152972_m1
CCL7	Hs00171147_m1	TLR9	Hs00370913_s1
CCL8	Hs04187715_m1	TLR10	Hs01935337_s1
CCL11	Hs00237013_m1	MyD88	Hs01573837_g1
CCL15	Hs00361122_m1	IRAK1	Hs01018347_m1
CCL18	Hs00268113_m1	IRF3	Hs01547283_m1
CCL19	Hs00171149_m1	IRF5	Hs00158114_m1
CCL20	Hs01011368_m1	Dectin-1	Hs01902549_s1
IL2RA	Hs00907779_m1	GAPDH	Hs03929097_g1

Supplementary Table S2: Clinico-demographic characteristics of males and females regarding Lean, Overweight, and Obese individuals

Metabolic Markers	Lean (n=10)		Overweight (n=19)		Obese (n=30)	
	Male (n=3)	Female(n=7)	Male(n=12)	Female(n=7)	Male (n=15)	Female (n=15)
Age (years)	42.00 ± 9.54	43.00 ± 8.33	44.17 ± 12.52	42.86 ± 9.08	45.13 ± 13.14	45.27 ± 13.28
Weight (kg)	69.93 ±13.81	59.93 ± 10.66	86.11 ± 4.86	68.34 ± 4.38	103.15 ± 12.51	85.81 ± 9.31
Height (m)	1.76 ± 0.12	1.61 ± 0.11	1.75 ± 0.05	1.55 ± 0.06	1.72 ± 0.07	1.56 ± 0.06
BMI (kg/m2)	22.44 ± 2.37	22.98 ± 2.52	28.20 ± 1.31	28.39 ± 1.06	34.65 ± 3.48	35.11 ± 3.10
Waist (cm)	86.33 ± 9.24	78.33 ± 13.82	99.40 ± 6.57	88.57 ± 8.04	114.13 ± 9.22	101.18 ± 12.60
Body fat (%)	22.23 ± 2.10	31.43 ± 5.22	28.84 ± 1.85	37.77 ± 1.85	36.03 ± 3.08	42.41 ± 2.34
FBS (mmol/L)	5.40 ± 0.87	4.79 ± 0.48	5.30 ± 0.82	5.20 ± 0.40	5.37 ± 0.64	5.37 ± 0.88
TGL (mmol/L)	0.60 ± 0.28	0.65 ± 0.24	1.27 ± 0.72	1.07 ± 0.49	1.32 ± 0.96	1.36 ± 0.74
Cholesterol (mmol/L)	4.63 ± 1.00	5.59 ± 1.10	4.94 ± 0.56	5.04 ± 1.00	4.97 ± 1.08	5.13 ± 1.20
HDL (mmol/L)	1.14 ± 0.29	1.93 ± 0.38	1.19 ± 0.25	1.40 ± 0.35	1.11 ± 0.24	1.22 ± 0.29
LDL (mmol/L)	3.20 ± 0.87	3.36 ± 1.02	3.21 ± 0.58	3.14 ± 0.85	3.25 ± 0.86	3.33 ± 1.14
HbA1c (%)	5.43 ± 0.42	5.76 ± 0.48	5.55 ± 0.54	5.39 ± 0.26	5.69 ± 0.58	5.71 ± 0.71
HOMA-IR	2.14 ± 0.62	1.03 ± 0.10	1.86 ± 1.22	1.50 ± 0.56	4.47 ± 3.88	4.32 ± 4.02
WBC	5.47 ± 2.30	5.62 ± 1.40	6.16 ± 1.60	6.03 ± 1.25	6.66 ± 2.25	6.32 ± 1.68

Supplementary Table S3: Correlation of Dectin-1 with all significantly correlated markers (Table 1) after adjustment to gender. Most of the correlations between dectin-1 and the studied factors, which were reported in Table 1, were also significant after adjusting for the gender effect. However, the correlation between decton-1 and CCL2, CXCL9, or RANTES showed marginal significance after adjusting for gender.

Inflammatory Markers	Pearson Correlation	
	r-value	*p-value*
**Immune and metabolic parameters**		
BMI	0.36	0.006
PBF	0.344	0.017
C Reactive Protein	0.369	0.023
Adiponectin	-0.508	0.002
Rantes	0.335	0.057
**Interleukins**		
IL-1b	0.311	0.042
IL-8	0.576	<0.0001
IL-10	0.56	<0.0001
IL-18	0.499	0.002
IL-23	0.55	<0.0001
TNF-α	0.484	0.0003
**CC chemokine ligands**		
CCL2	0.252	0.066
CCL3	0.543	<0.0001
CCL5	0.53	0.0002
CCL7	0.505	0.0001
CCL18	0.564	<0.0001
CCL20	0.635	<0.0001

Inflammatory Markers	Pearson Correlation	
	r-value	*p-value*
**Cytokine/chemokines receptors**		
CCR1	0.442	0.0008
CCR2	0.463	0.001
CCR5	0.675	<0.0001
**CXC chemokines ligands**		
CXCL9	0.256	0.055
CXCL10	0.427	0.001
CXCL11	0.439	0.001
**TLRs and downstream signaling markers**		
TLR2	0.65	<0.0001
TLR7	0.475	0.0002
TLR8	0.638	<0.0001
TLR10	0.422	0.002
MyD88	0.389	0.003
IRAK1	0.355	0.008
IRF5	0.477	0.003
**Monocyte/macrophage surface markers**		
CD16	0.733	<0.0001
CD68	0.493	0.0001
CD86	0.68	<0.0001
CD163	0.604	<0.0001

Supplementary Figure S2. **Correlation of AT dectin-1 gene expression with that of various cytokines/chemokines and their cognate receptors.** In the studied cohort, we performed association studies between the levels of dectin-1 transcripts and that of different cytokines, chemokines, and their receptors as well as the TLR signaling cascade and cellular surface markers.

Interleukins

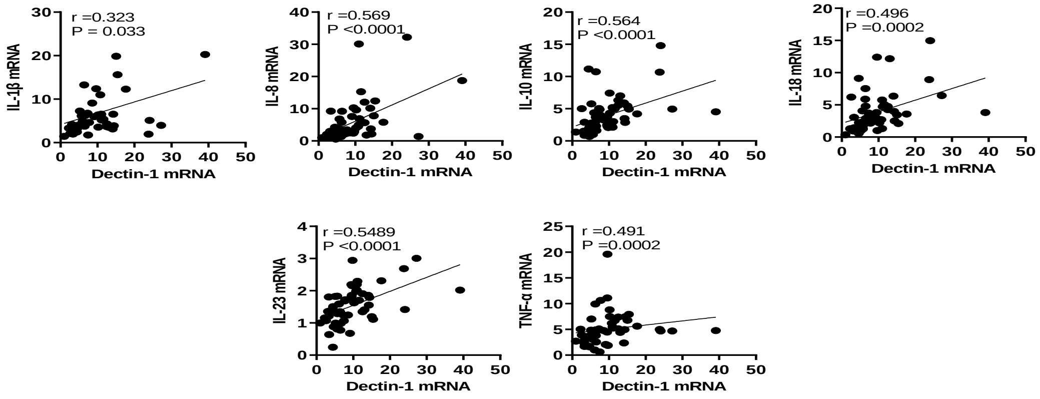

Cytokine/chemokines receptors

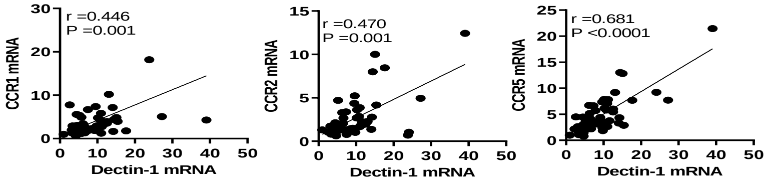

CC chemokine ligands

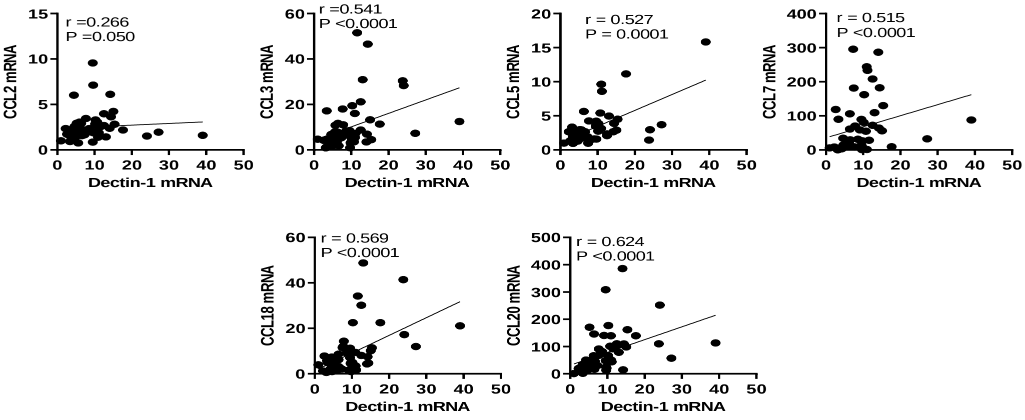

CXC chemokines ligands

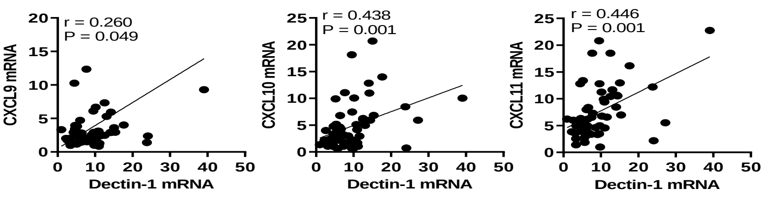

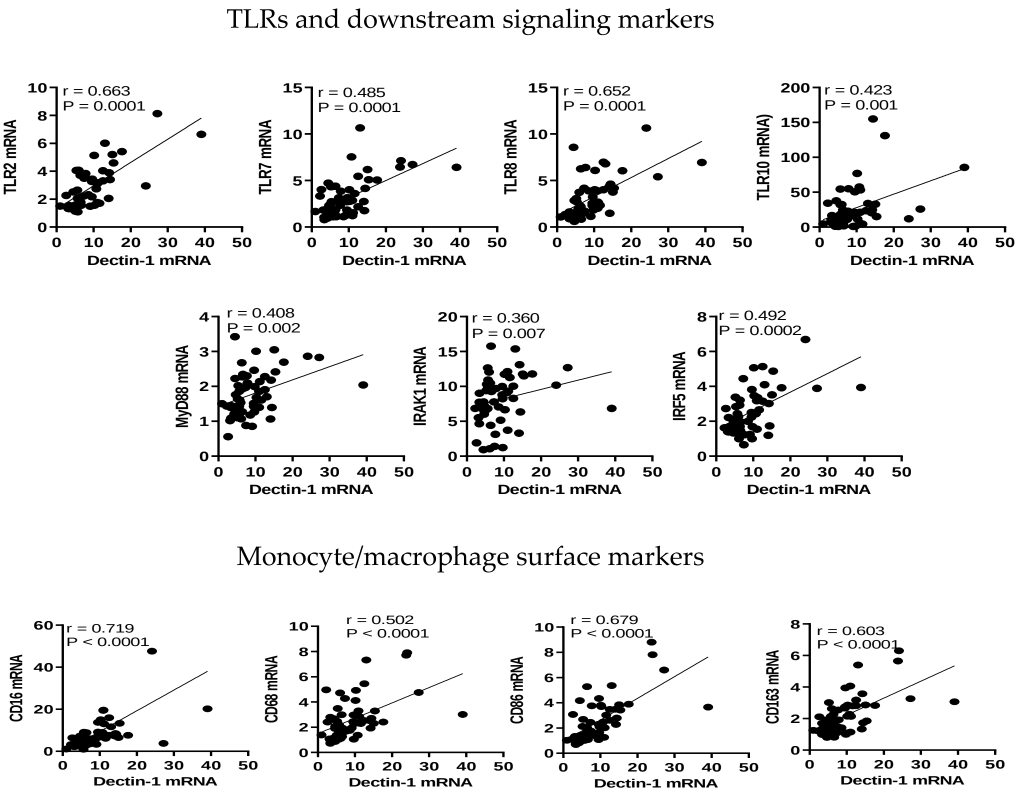

TLRs and downstream signaling markers
r = 0.663
P = 0.0001
TLR2 mRNA
Dectin-1 mRNA
r = 0.485
P = 0.0001
TLR7 mRNA
Dectin-1 mRNA
r = 0.652
P = 0.0001
TLR8 mRNA
Dectin-1 mRNA
r = 0.423
P = 0.001
TLR10 mRNA)
Dectin-1 mRNA
r = 0.408
P = 0.002
MyD88 mRNA
Dectin-1 mRNA
r = 0.360
P = 0.007
IRAK1 mRNA
Dectin-1 mRNA
r = 0.492
P = 0.0002
IRF5 mRNA
Dectin-1 mRNA
Monocyte/macrophage surface markers
r = 0.719
P < 0.0001
CD16 mRNA
Dectin-1 mRNA
r = 0.502
P < 0.0001
CD68 mRNA
Dectin-1 mRNA
r = 0.679
P < 0.0001
CD86 mRNA
Dectin-1 mRNA
r = 0.603
P < 0.0001
CD163 mRNA
Dectin-1 mRNA

Supplementary Figure S3. Representative confocal microscopy images for colocalization of dectin-1 and CD64 (A) and CD163 (B). Adipose tissues from lean, overweigh and obese individuals were incubated with antibodies directed against dectin-1, CD64 or CD163 and then with secondary antibodies Alexa fluor 647 or 488, as indicated. Nuclei were stained with DAPI. (C) The intensity of fluorescence for CD64 and CD163 were measured in all tested slides.

A

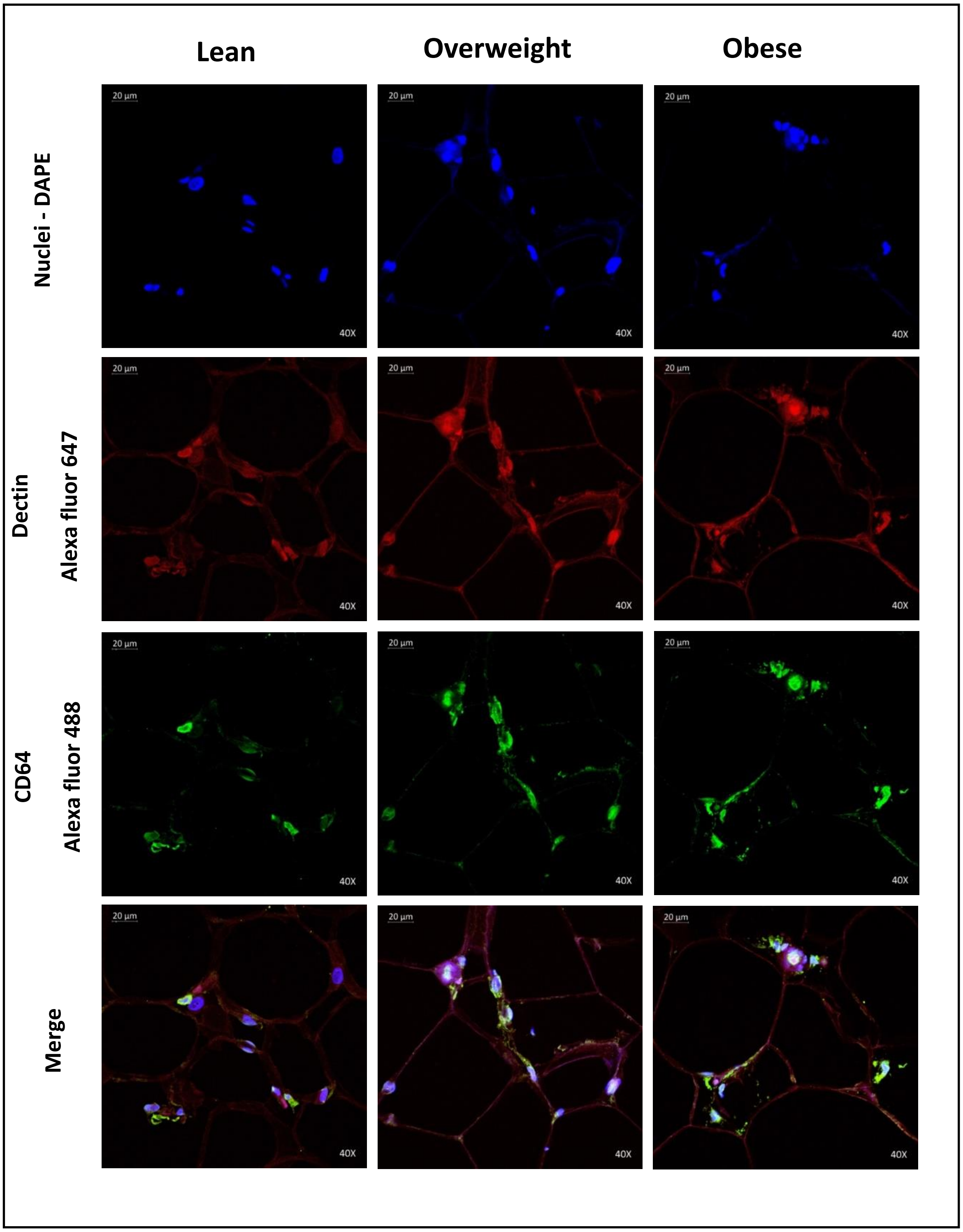

B

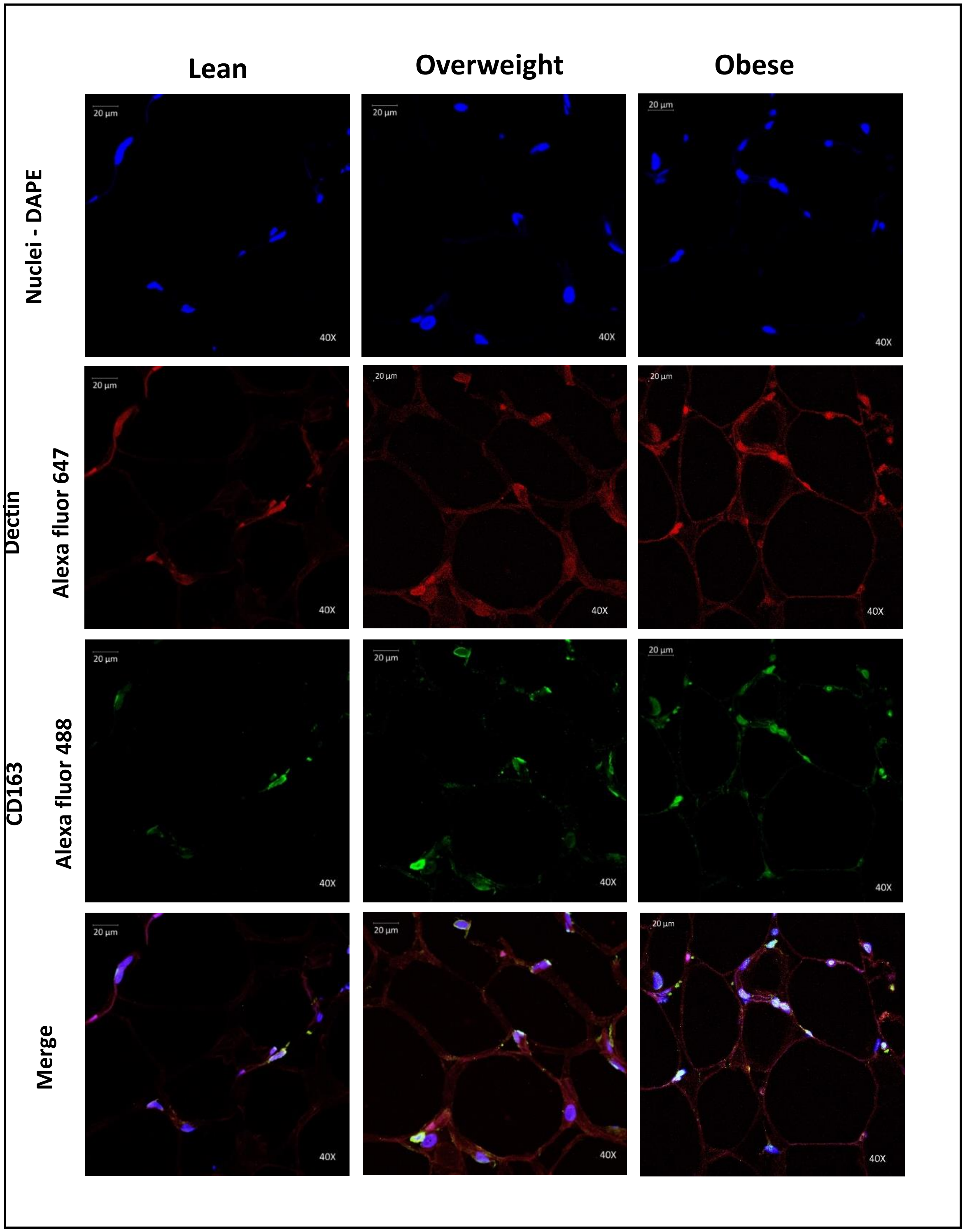
Lean
Overweight
Obese
Nuclei - DAPE
Dectin
Alexa fluor 647
CD163
Alexa fluor 488
Merge
20 µm
40X

C

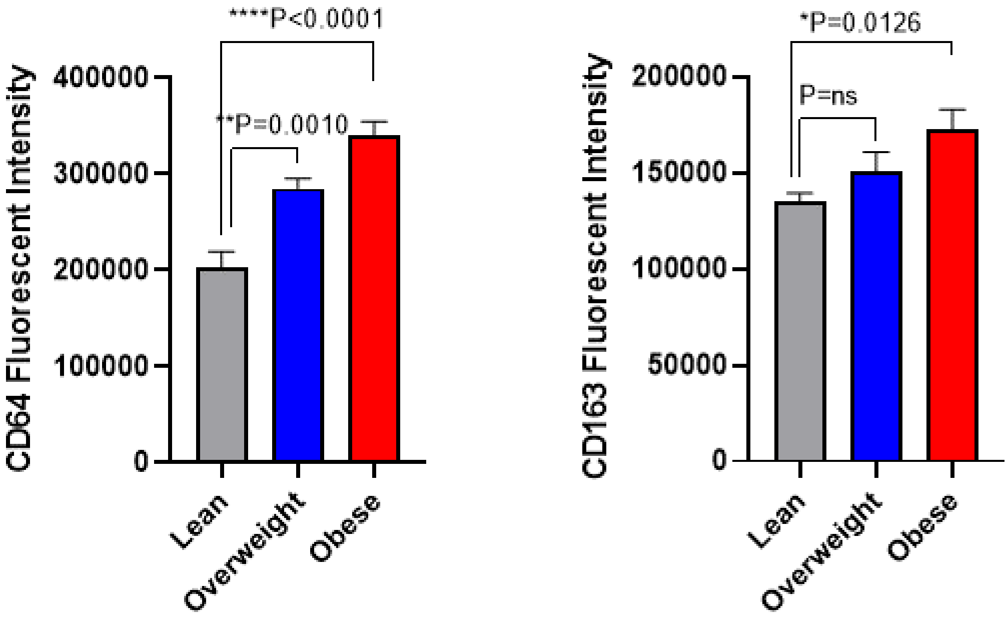
****P<0.0001
**P=0.0010
CD64 Fluorescent Intensity
400000
300000
200000
100000
0
Lean
Overweight
Obese
*P=0.0126
P=ns
CD163 Fluorescent Intensity
200000
150000
100000
50000
0
Lean
Overweight
Obese
